# Engraftment of strictly anaerobic oxygen-sensitive bacteria in irritable bowel syndrome patients following fecal microbiota transplantation does not improve symptoms

**DOI:** 10.1080/19490976.2021.1927635

**Published:** 2021-06-01

**Authors:** Patrick Denis Browne, Frederik Cold, Andreas Munk Petersen, Sofie Ingdam Halkjær, Alice Højer Christensen, Stig Günther, Lars Hestbjerg Hansen

**Affiliations:** a Department of Plant and Environmental Sciences, University of Copenhagen, Frederiksberg, Denmark; b Gastrounit, Medical Division, Copenhagen University Hospital Hvidovre, Hvidovre, Denmark; c Department of Gastroenterology, Aleris-Hamlet Hospitals Copenhagen, Soeborg, Denmark; d Department of Clinical Microbiology, Copenhagen University Hospital Hvidovre, Hvidovre, Denmark

**Keywords:** Fecal microbiota transplantation, FMT, irritable bowel syndrome, gut microbiome, dysbiosis, engraftment, diversity, anaerobic bacteria, amplicon sequence variants, bowel cleansing

## Abstract

Dysbiosis of the gut microbiome has been correlated with irritable bowel syndrome (IBS). Fecal microbiota transplantation (FMT) is being explored as a therapeutic option. Little is known of the mechanisms of engraftment of microbes following FMT and whether the engraftment of certain microbes correlate with clinical improvement in IBS. Microbiome data, from a previously reported placebo-controlled trial of treatment of IBS with FMT or placebo capsules, were used to investigate microbial engraftment 15 days, 1, 3 and 6 months after treatment through assessment of gains, losses and changes in abundance of amplicon sequence variants (ASVs) and microbial diversity (CHAO-1 richness) between the FMT group and the placebo group. These data were compared to changes in IBS Symptom Severity Scores (IBS-SSS). Twelve days of treatment with 25 daily multi-donor FMT capsules induced significant short- and long-term changes in the recipients’ microbiomes for at least 6 months, with persistent engraftment of a variety of anaerobic bacteria from keystone genera, such as *Faecalibacterium, Prevotella* and *Bacteroides* and increased microbial diversity, particularly in patients with low initial diversity. FMT recipients lost ASVs after treatment, which was seen to a much lesser extent in the placebo group. No ASVs increased to a greater extent between FMT responders and non-responders following treatment. Major long-term changes, lasting for at least 6 months, in the gut microbiomes of IBS patients are seen following treatment with FMT capsules. None of these changes correlated with clinical improvement. The relationship between the microbiome and the etiology of IBS still remains unsolved.

## Introduction

Research in recent years, driven by improved methods of culture-independent microbial community analysis, has revealed that the gut microbiome is an important element of human health and well-being.^1^ It plays important roles in modulating the immune system, digesting food, hormone regulation, and pathogen exclusion.^2^ An altered gut microbiome, often referred to as gut dysbiosis, has been reported to be correlated with a wide variety of conditions including gut diseases such as irritable bowel syndrome (IBS), Inflammatory Bowel Diseases (IBD)and recurrent *Clostridioides difficile* infection (rCDI), but also in non-gastrointestinal diseases such as obesity, type 2 diabetes and mental disorders.^3–8^

Modulating the gut microbiome is thus seen as an important tool to improve human health (e.g. to cure a disease). Fecal microbiota transplantation (FMT) is one strategy to actively modulate the gut microbiome. It aims to introduce a balanced conglomerate of microorganisms (bacteria, viruses, fungi, parasites and archaea) from a healthy fecal donor into a patient, potentially treating the disease proposedly caused by a gut dysbiosis.

Although FMT is very effective in treating rCDI, where it is superior to antibiotics,^9^ FMT has demonstrated inconsistent outcomes when used to treat other dysbiosis-related conditions and is so far not a recommended intervention for any disease other than rCDI.^10^

IBS is characterized by recurrent abdominal pain and further symptoms, such as altered stool frequency, diarrhea, bloating or constipation, and it affects 10–15% of the global population.^11,12^ The etiology behind IBS is not fully understood, but low-grade intestinal inflammation caused by an altered gut microbiota has been correlated with IBS.^3,13^ The fact that there is a considerable risk of developing IBS following an episode of infectious gastroenteritis also implicates a correlation with the gut microbiome.^14^ The gut microbiome of patients with IBS is characterized by lower microbial diversity and changed abundances of certain bacteria compared to healthy controls. However, not all studies report these correlations and the differences in abundances of bacteria vary between study populations.^15,16^ Whether these changes are a cause or a consequence of the disease is still not fully understood, highlighting a major knowledge gap in the relationship between the gut microbiome and IBS.

Consequently, it has been proposed that FMT from healthy donors could alter the dysbiotic microbiota of IBS patients toward a “normal” eubiotic state. Different studies investigating the treatment of IBS using FMT have shown divergent results in placebo-controlled randomized clinical trials (RCTs).^17–20^

The dataset used in this work comes from a previously published RCT testing 12 days of daily multi-donor FMT or placebo (1:1 ratio) capsules preceded by a bowel cleansing in 52 IBS patients.^21^ The previous publication focused on the clinical effects of the treatment. The patients experienced improved symptoms measured by the irritable bowel syndrome symptom severity score (IBS-SSS) following treatment both in the FMT and placebo groups. Surprisingly, this improvement was strongest in the placebo-group at the primary endpoint after 3 months. Microbiome changes were addressed in broad terms, reporting that fecal microbial biodiversity increased and the microbiomes became more similar to the donors after treatment in the patients receiving FMT capsules.

This work aims to thoroughly address the dynamics of engraftment at various time-points following FMT treatment and whether the changes were correlated with clinical effects. This was done through re-analysis of the sequencing reads using a more recent approach of denoising reads into amplicon sequence variants (ASVs)^22^ and by assessing engraftment at four different time-points (namely 2 weeks, 1 month, 3 months and 6 months) following treatment. To address whether microbiome changes were correlated with clinical effects, we analyzed whether patients who exhibited a clinical response (responders) and those who did not (non-responders) experienced different changes following FMT.

## Results

Fecal samples were collected at five different timepoints (baseline, 15 days, 1, 3 and 6 months) from the 52 included patients. Following amplicon sequencing of the patients’ 216 available samples, and the donors’ 4 samples, a total of 7,279,663 sequencing reads were denoised into 2165 ASVs. A minimum of 6,483 to a maximum of 113,847 reads per sample were assigned to ASVs. These ASVs were assigned to 14 phyla, 27 classes, 34 orders, 51 families, 103 genera, and 186 species. Clinical symptoms were assessed through IBS-SSS^23^ and response was defined as a decrease ≥50 in IBS-SSS after 3 months compared to baseline. There was no difference in clinical symptoms or microbiome diversity between FMT and placebo responders and non-responders at baseline (Table 1).

**Table 1.** Baseline characteristics of FMT and Placebo responders and non-responders

	FMT responders (*n* = 8)	FMT non-responders (*n* = 13)	*P*-value	Placebo responders (*n* = 19)	Placebo non-responders (*n* = 5)	*P*-value
Age median (range)	38.5 (19–55)	35.5 (22–57)	1.000	33 (21–50)	38 (18–55)	0.618
Sex F/M	6/2	9/4		12/7	4/1	
IBS-M/IBS-D/IBS-C	4/3/1	5/3/5		6/6/7	2/2/1	
IBS-SSS median (range)	370.5 (211–477)	396 (171–480)	0.971	350 (239–485)	349 (205–375)	0.177
Fecal alpha diversity (CHAO-1-richness) mean (SD)	585 (95)	529 (135)	0.321	594 (113)	559 (121)	0.544

FMT, Fecal Microbiota Transplantation; F, Female; M, Male; IBS-M/IBS-D/IBS-C, Irritable bowel syndrome mixed/diarrhea/constipation.

The majority of patients were categorized with severe disease at baseline based on IBS-SSS of 75 to 175, 175 to 300 and > 300, respectively, indicating mild, moderate and severe cases of disease (Table 2).^23^ As reported in the primary publication presenting the clinical data from this trial, the patients in the placebo group improved the most at the primary endpoint after 3 months.^21^ Further indicating that the FMT treatment did not improve the symptoms of the patients as much as the placebo treatment is the fact that still almost half the patients in the FMT group (47.6%) reported severe symptoms after 3 months compared to 29.1% in the placebo group.

**Table 2.** Severity of IBS at various timepoints following treatment

	FMT (*N* = 21)	Placebo (*N* = 24)
**Baseline**: Remission Mild Moderate Severe	0 0 7 14	0 0 5 19
**One month**: Remission Mild Moderate Severe	0 6 6 9	1 9 7 7
**Three months**: Remission Mild Moderate Severe	1 4 6 10	0 13 4 7
**Six months**: Remission Mild Moderate Severe	1 3 5 12	2 6 9 7

Severity of Irritable Bowel Syndrome based on Irritable Bowel Syndrome Symptom Severity Scores (IBS-SSS). Less than 75 indicates remission, 75 to 175 indicates mild disease, 175 to 300 indicates moderate disease and more 300 indicates severe disease. IBS, Irritable Bowel Syndrome; FMT, Fecal Microbiota Transplantation.

### FMT treatment increased fecal microbiome diversity both short- and long-term

**Figure 1.**
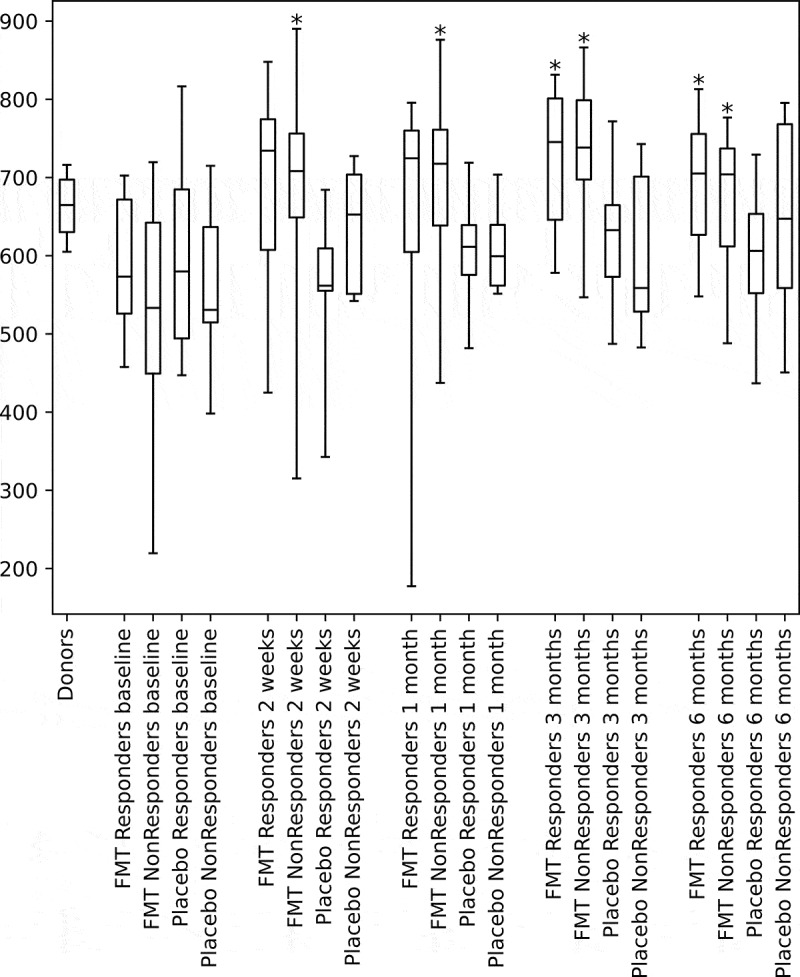
Change in fecal microbial diversity after FMT and Placebo treatment. Alpha diversity (Chao1) is shown for the donors and treatment groups at baseline and at all post-treatment time-points. Asterisks indicate significant difference from baseline within each treatment group (*p* < .05). Horizontal lines indicate medians, boxes indicate interquartile ranges and whiskers indicate ranges. FMT, Fecal Microbiota Transplantation

In the FMT-treated patients, diversity (CHAO1-richness)^24^ was seen to significantly increase, relative to baseline, at all following time-points including the last follow-up after 6 months (Figure 1). The increases in diversity relative to baseline were significant in the FMT group, when compared to the placebo group, at all time-points (Supplementary file, Table S1a and S1b).

There were no significant differences between the changes in diversity between responders and non-responders at any time-point in the FMT-treated patients. Neither were the changes in diversity from baseline significantly different between the placebo responders and the placebo non-responders (0.12 ≤ *p*≤ 0.61). Changes in diversity did not significantly correlate with changes in IBS-SSS, according to Spearman-rank correlations (according to the criteria: *p*≤ 0.05, R^2^ ≥ 0.15) at any timepoint whether looking at all patients combined or within the patient groups (FMT responders, FMT non-responders, placebo responders or placebo non-responders) (Supplementary file, Figure S1a and S1b). This held true using Chao1-richness and two other measures of diversity, namely effective Shannon diversity and effective Simpson diversity.^25,26^

### Microbial diversity increased the most in patients with low baseline diversity

**Figure 2.**
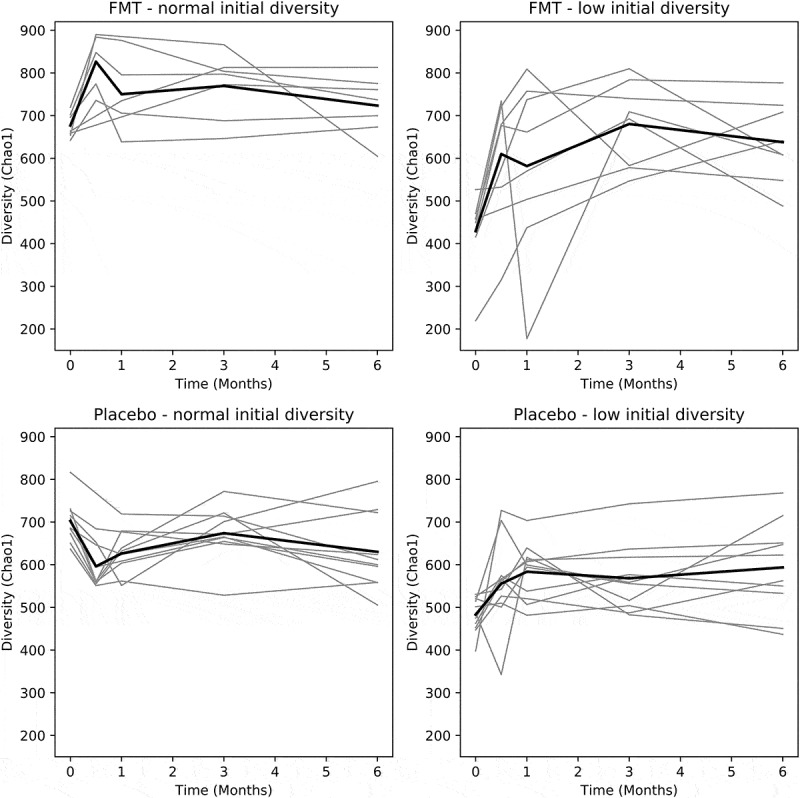
Change in diversity in patients with initial low and normal diversities. Grey lines represent individual patients and black lines represent average diversity of each group; FMT, Fecal Microbiota Transplantation

Patients were classed as having low- or normal initial diversity based on their baseline diversities (see methods section). As a result of these definitions, ten FMT patients were classified as having normal initial diversity and seven were classified as having low initial diversity. In the placebo group, the numbers of patients assigned to the normal and low initial diversity groups were 13 and 4, respectively. Four FMT and seven placebo patients were unclassified, falling between the two cutoffs. FMT patients with low initial diversity and FMT patients with normal initial diversity experienced increases in diversity at all time-points (Figure 2). The changes in diversity from baseline were, on average, greater at all time-points in the FMT group with low initial diversity compared to the group with normal initial diversity. These changes between the FMT groups with low and normal diversity at baseline were statistically significant after 3 and 6 months (*p*= .009 and 0.002, respectively). In the placebo groups, the average changes in diversity were always positive for those patients with low initial diversities and negative for those with normal initial diversities without these changes from baseline being significant.

### FMT-treated patients’ microbiota became more similar to the donors’, both short- and long-term

To evaluate time-dependency of engraftment, we analyzed the generalized unifrac distances (β-diversity) of the patients to the donors, which became significantly smaller for the FMT responders after 3 and 6 months, and for the FMT non-responders at all time-points (Supplementary file, Table S2). The placebo responders were significantly more dissimilar to the donors after 15 days, 1 month and 3 months, but not after 6 months. The placebo non-responders did not change significantly in generalized unifrac distances to the donors.

### FMT altered abundances of certain ASVs among diverse types of bacteria

Comparing ASV abundances between patient groups at different time-points revealed that FMT treatment drove statistically significant changes in ASV abundances, while no systemic changes in ASV abundances were associated with clinical response versus non-response (Supplementary file, Table S3). At baseline, no differences were observed between any patient groups. Following treatment, at all time-points there were between 66 and 106 ASVs with altered abundance between the FMT group and the placebo group, and between 60 and 74 ASVs different between the FMT responders and the placebo responders. No ASVs were differently abundant between the FMT non-responders and the placebo non-responders probably due to the low statistical power stemming from there being only five placebo non-responders. Comparing clinical responders with non-responders revealed no significant ASV abundance differences regardless of whether we look at these groups overall or broken down into respective FMT and placebo groups. Where ASV abundance differences occurred between groups, the ASVs were predominantly differently abundant at more than one but not at all time-points (Supplementary file, Figure S2).

**Figure 3.**
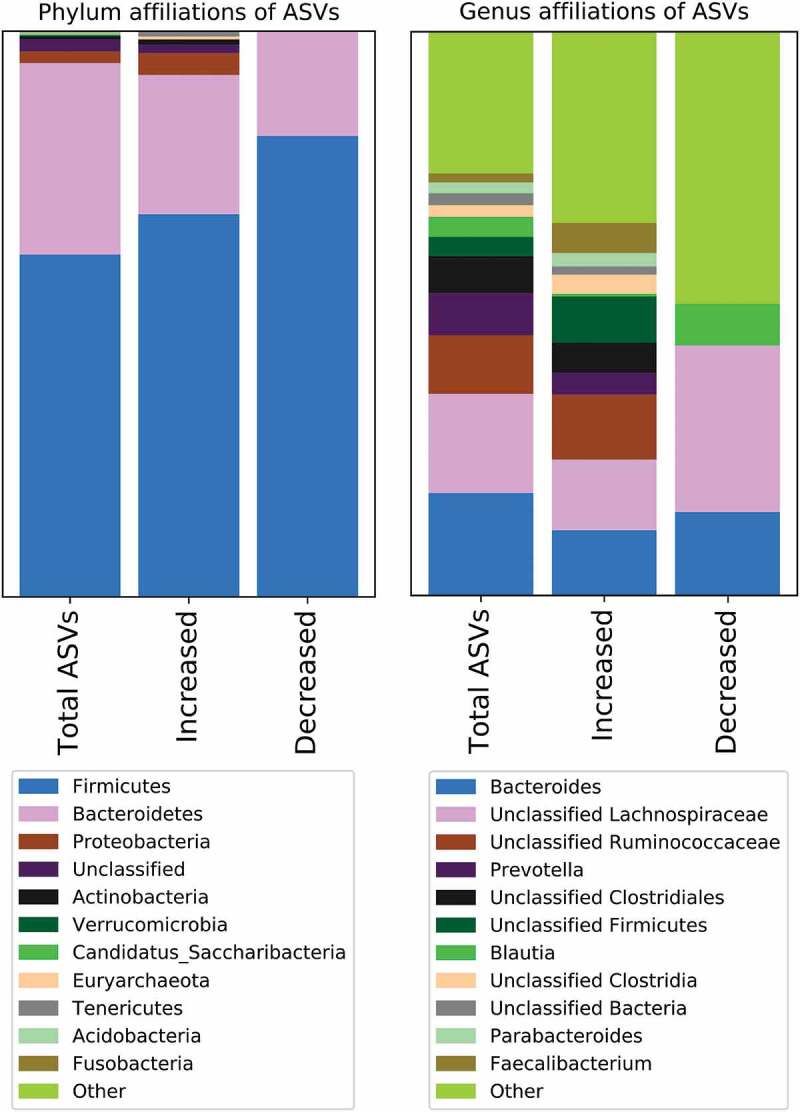
Phylum and genus of bacteria with increased/decreased relative abundance after FMT. The colored areas indicate the proportion of ASVs, among the total number of ASVs either detected (Total ASVs), increased in abundance in FMT patients, or decreased in abundance in FMT patients, assigned to the phyla or genus indicated below. The increase and decrease columns are based on all post-baseline samples from the FMT-treated patients, when compared to placebo treated patients

The changes in abundances were distributed among taxonomically distant ASVs (Figure 3). Among the eight groups (two treatment comparisons at four time-points) showing changes in ASV abundances, ASVs from between 4 and 7 phyla, 9 and 13 orders, and 21 and 26 genera were increased per group, while ASVs from up to 2 phyla, 2 orders and 8 genera decreased in abundances per group. The genera represented among the ASVs increasing in abundance in the FMT group relative to the placebo group include *Faecalibacterium, Bacteroides, Prevotella* and *Lactobacillus*.

There were no correlations between the abundances of any ASV and symptoms assessed through IBS-SSS according to Benjamini–Hochberg corrected Pearson correlation tests (data not shown) (*p*> .05).

### FMT induces both significant engraftment and loss of bacteria

The application of criteria to account for sequencing depth disparities (Supplementary file, Figure S3) and natural ASV abundance fluctuations when assessing gains and losses of ASVs following treatment (see methods section) indicated that the FMT patients gained and lost significantly more ASVs than the placebo patients (*p*< .05). These gains and losses of ASVs are on a per patient basis, which is in contrast to the systemic changes in ASV abundances examined in the section above. The FMT patients gained a median of 81 ASVs and lost a median of 38 ASVs, which were both significantly more than the respective median figures of 11 gains and 5 losses for the placebo group (Supplementary file 2). When comparing the FMT responders with the FMT non-responders, there was no significant difference in gains (both medians being 81 ASVs gained, *p*= .96) while there was a significant difference between the loss rates (44 (FMT responders) vs 25 (FMT non-responders), *p*= .01). There were no significant differences observed in the gain or loss rates between the placebo responders and the placebo non-responders (*p*= .83 and *p*= .94, respectively).

Among the 20 FMT patients (from whom sufficient data were available), one specific ASV was gained by 12 different patients and another by 11 different patients (Supplementary file 2). Both were assigned to the species *Phascolarctobacterium succinatutens*. Gain rates in the FMT group were significantly above the background levels seen in the placebo group for some keystone human gut microbiome genera, such as, *Bacteroides, Prevotella* and *Faecalibacterium* (Table 3). The highest number of patients from whom any particular ASV was lost following treatment was the same, at six patients, for both the FMT and placebo patients (Supplementary file, Figure S4). However, there were 40 ASVs lost from three or more FMT patients while only 10 ASVs were lost from three or more placebo patients. Loss rates were above background (placebo group) in the FMT patients for the *Bacteroides* genus, but not for *Prevotella* nor *Faecalibacterium*.

**Table 3.** Engraftment and loss of ASVs driven by FMT

Genus	Num. ASVs	FMT gains	Placebo gains	FMT loss	Placebo loss
*Bacteroides*	394	126 (247) [8]	35 (38) [2]	147 (214) [4]	27 (28) [2]
*Prevotella*	163	51 (136) [7]	19 (24) [3]	0 (0) [0]	2 (2) [1]
*Faecalibacterium*	34	27 (59) [7]	17 (18) [2]	0 (0) [0]	1 (3) [3]
*Blautia*	77	39 (58) [4]	27 (38) [3]	8 (12) [3]	7 (8) [2]
*Ruminococcus*	39	11 (33) [7]	5 (5) [1]	12 (15) [2]	13 (23) [3]
*Phascolarctobacterium*	6	3 (33) 12	0 (0) [0]	1 (5) [5]	0 (0) [0]
*Parabacteroides*	41	16 (31) [6]	0 (0) [0]	5 (7) [2]	1 (1) [1]
*Sutterella*	6	3 (25) [9]	1 (1) [1]	0 (0) [0]	2 (3) [2]
*Alistipes*	20	11 (23) [8]	3 (3) [1]	2 (2) [1]	0 (0) [0]

The most frequently changing (gains plus losses) genera are shown. The number of ASVs assigned to each genus is shown (Num. ASVs). The numbers indicate (i) the number of ASVs gained or lost, (ii) in parentheses, the total number of gains or losses for that genus among the patient group and (iii) in square brackets, the highest number of patients in which any of the ASVs were gained or lost.

**Figure 4.**
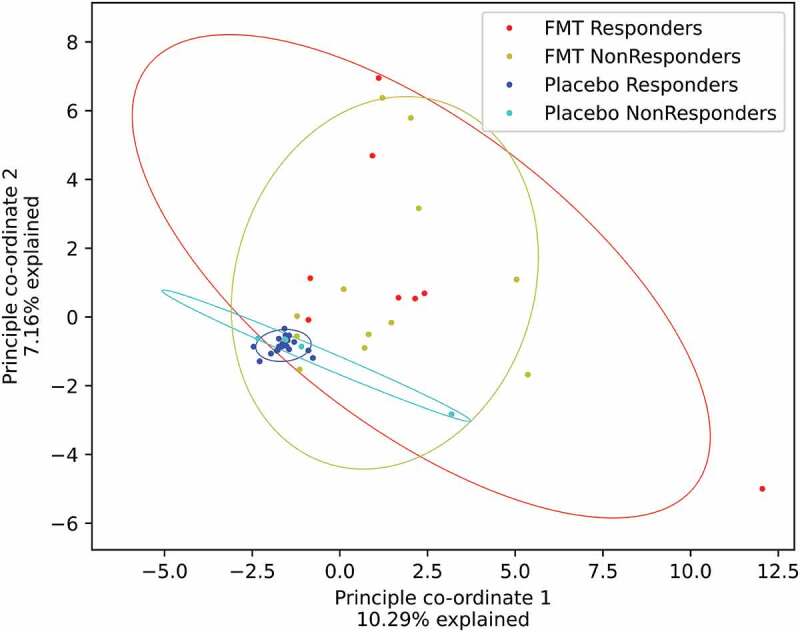
Principle coordinate analysis of ASV gains and losses in the FMT and Placebo responders and non-responders

The FMT patients had considerable individuality in their gain and loss profiles (Figure 4). The gains and losses of ASVs followed significantly different trends within the FMT group compared to within the placebo group (Supplementary file, Figure S5) (*p*< .001). There was no significant difference between FMT responders and FMT non-responders (*p*= .15). No single ASV was gained or lost predominantly in the FMT responders or FMT non-responders.

### Bacteria derived from the donors were found in the FMT patients after treatment

Another approach to examine the engraftment of donor microbes in patients was to use software implementing a Bayesian algorithm^27^ which estimates the proportions of a patient’s post-treatment microbiota that can be attributed to coming from either their pre-treatment microbiota or from the donors’ microbiota. This approach showed that higher proportions of the FMT patients’ (58–68%) than the placebo patients’ (25–36%) (*p*≤ 0.05) post-treatment microbiota were inferred to have come from the donors at all post-treatment time-points. Importantly, there were no differences in engraftment between the FMT responders and the FMT non-responders, except for more of one particular donor’s microbiota (Donor 2) explaining a higher portion of the FMT responders’ microbiota than the FMT non-responders at the six-month time-point only.

**Figure 5.**
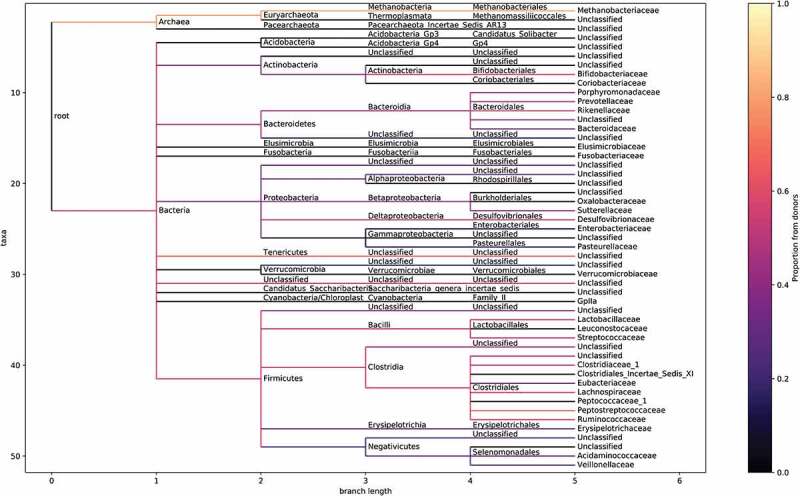
Proportions of different bacteria/archaea derived from the donors in the FMT patients. In each FMT patient’s sample for the relevant time-point (3 months), the proportions of each ASV attributed to coming from the donors was inferred using sourcetracker2. These proportions were all averaged to get an engraftment score for that node. Similarly, an engraftment score was also calculated for the placebo group. The score associated with the placebo group was used as a floor to rescale score assigned to the FMT group

Engraftment occurred to different degrees in different clades, and variations in engraftment occurred within clades. Some phyla, such as *Verrucomicrobia, Acidobacteria* and *Fusobacteria*, have very low or even no engraftment, though these phyla are numerically less important. Other phyla, such as the *Firmicutes, Bacteroidetes, Actinobacteria* and *Euryarchaeota*, have moderate to high engraftment, though these tend to have sub-lineages that do not engraft as strongly as others, if at all (Figure 5).

## Discussion

Based on this follow-up on a previously published RCT treating IBS patients with FMT or placebo, we can conclude that 12 days of multi-donor FMT delivered through capsules induces long-term changes in the recipients’ microbiomes. FMT leads to the engraftment of numerous microbes with a broad taxonomic distribution in FMT patients, including strictly anaerobic bacteria, and also induces alterations of the recipients’ microbiomes with significant losses of microbes. There were no apparent differences in any ASV levels between the FMT recipients that exhibited a clinical response and those who did not. The changes in the microbiome were not correlated with clinical effects in this group of IBS patients highlighting that the understanding of the role of the microbiome in IBS still remains unsolved.

The changes in the FMT recipients’ microbiomes were long term, with significant changes persistent for at least 6 months, which was the last follow-up time-point of this study. This indicates that FMT delivered through capsules can drive major, widespread and lasting changes in the human intestinal microbiome. We were not able to correlate engraftment nor removal of certain ASVs with positive responses by the patients. However, it was evident from this study that: (i) FMT treatment resulted in a fast and long-lasting increase in fecal microbiome diversity, observable at all post-treatment time-points, (ii) microbial diversity increased the most in patients with low baseline diversity and (iii), FMT-treated patients’ microbiota became more similar to the donors’, evident at all time-points throughout the 2 week to 6 month post-treatment follow-up period, and (iv) it is possible to transfer strictly anaerobic and oxygen-sensitive bacteria through FMT capsules.

We assessed engraftment of bacteria after FMT in a variety of different ways in order to identify FMT-driven changes in the microbiomes at different scales. The long-term engraftment after treatment with FMT capsules is in concordance with data from other populations. Both healthy volunteers^28^ and patients with rCDI experience long-term engraftment for at least a year after treatment with capsule FMT.^29^ FMT altered the abundances of and drove the gain and loss of ASVs of several keystone genera including *Faecalibacterium, Prevotella*, and *Bacteroides* (Figure 3 and Table 3). *Faecalibacterium prausnitzii* is a propionate-producer and its loss is associated with inflammatory conditions.^30^ *Prevotella copri* has been both positively and negatively associated with human health and has been reported to be more abundant in non-Westernized populations.^31–33^ *Bacteroides* species play important roles in human nutrition, producing volatile fatty acids, and the ratio of *Bacteroides* to *Prevotella*, which are considered to be antagonistic, is relevant to weight control in humans.^34^ It is widely known that the majority of fecal microorganisms are anaerobic, but it has been shown elsewhere that the members of the Firmicutes, especially *Faecalibacterium* and *Megamonas*, lose viability upon oxygen exposure during fecal processing much more quickly relative to other anaerobic genera such as *Bacteroides* and *Parabacteroides*.^35^ Results here indicate that ASVs of both relatively oxygen-sensitive microbes (*Faecalibacterium* (Table 2) and *Megamonas* (not shown)) and relatively oxygen-resistant genera, e.g. *Bacteroides* and *Parabacteroides*, engraft in FMT patients significantly above the levels at which they are seen to be acquired from natural sources by placebo patients. This verifies that steps taken to minimize oxygen intrusion (e.g. flushing with argon) during the preparation of the FMT capsules were sufficiently effective.

The FMT patients in our study lost much higher numbers of ASVs than the placebo patients did, which has not been reported before. This suggests that the establishment of donor microbes, or bacteriophages present in the FMT treatment, displaced microbes that were present pre-treatment. FMT has recently been reported to be effective in the treatment of multidrug resistant bacteria.^36^ This effect is proposedly caused by both engraftment of beneficial microbes and displacement of harmful bacteria caused by FMT. We found that the FMT-responders lost more ASVs, than the FMT non-responders, which also might indicate that potential beneficial effects of FMT can be attributed to the displacement of some bacteria following the treatment.

In the placebo group, the participants with low baseline diversity tended to show a statistically insignificant increase in their diversity when compared to the participants with normal baseline diversity. This is possibly related to normal fluctuations in the human microbiota where those with low initial diversity were simply near the trough of a wave and thus more likely to show an increase at later sampling times. Over the whole placebo group, the diversity did not change from baseline suggesting that the bowel cleansing did not alter diversity in such a way that was evident after 2 weeks. The fact that no particular ASVs were lost from most of the placebo patients cannot be used to conclude that loss of some bacteria or broad microbiome changes in individual patients did not happen after bowel cleansing. The fact that 19 out of 24 (79.2%) placebo patients were defined as responders, which is higher than the expected 30–40% placebo effect,^37^ indicates that the bowel cleansing might have induced a positive clinical effect. This is in contrast to proposed potentially harmful effects of bowel cleansing where a reduction in bacterial gut microbiota diversity for up to 4 weeks has been reported,^38^ which is in contrast to our finding of no decrease in diversity either short or long term. Since constipation is often a part of the disease course of many IBS patients, a bowel cleansing might induce beneficial effects in some patients. Interestingly, the highest degree of improvement in clinical symptoms after 3 months was in the IBS-Constipation subgroup.^21^ No other studies have reported the effects of bowel cleansing in IBS patients and we urge researchers to investigate this in future studies.

The relationship between the microbiome and the etiology of IBS still remains unsolved.^3,16^ The term gut dysbiosis is often used in relation to IBS and other diseases while the term itself is not clearly defined and is probably different from disease to disease.^39,40^ Typically, gut dysbiosis is described, but not strictly defined, as low bacterial diversity, and changed proportions of certain bacteria mainly based on correlations and not interventional studies.^40,41^ Based on the current research, it is not possible to conclude if FMT treatment is beneficial for IBS patients since the results of RCTs are mixed.^17^ The two published RCTs testing FMT capsules have been unsuccessful,^19,21^ while FMT delivered through endoscopy (both upper and lower) has been beneficial in some, but not all, studies, when compared to placebo.^18,42,43^ However, many studies have used autologous FMT as placebo treatment and hence the true effect of the donor derived allogenic FMT is difficult to distinguish. As we report no beneficial effects of increasing gut microbiome diversity and Goll et al.^44^ reported response only in patients, that did not increase their diversity, based on microbiome data from a study of FMT treatment to IBS patients delivered through colonoscopy,^45^ simply to increase gut microbiome diversity following FMT cannot be used as a marker of beneficial effects.

Since neither changes in the gut microbiome diversity nor a proposed dysbiosis index, used by El-Salhy et al. in another RCT of successful FMT treatment in IBS subjects,^18,46^ correlated with clinical improvements, it can be concluded that the current understanding of gut dysbiosis in IBS is still inadequate, and is why further studies are warranted.

### Strengths and weaknesses

The primary strength of this study is in particular, the placebo-controlled design that gives us the opportunity to quantify the changes in the microbiome caused by FMT by accounting for natural and bowel-cleansing driven fluctuations in human microbiota. Further, we performed detailed investigations into engraftment utilizing a variety of complementary approaches. This provides detailed insight into the effects of encapsulated FMT treatment on recipients’ microbiota, documenting previously unknown patterns of microbial engraftment and displacement across the prokaryotic tree of life. There are also limitations to our analyses. Firstly, all the available samples were fecal samples. Intestinal biopsies would have given a more exact description of the changes in the tissue, where the microbiome is more likely to be involved in the disease pathology. Further, there is no general consensus regarding how to define low and normal diversity. In this study, we have tried to define low and normal diversity using the healthy FMT donors’ diversities as comparators, which makes the results difficult to compare to other studies. The use of a definition of clinical response as at least a 50-point reduction in IBS-SSS after 3 months compared to baseline might not be ideal since many patients can still have severe symptoms of IBS while being defined as responders.^23^ Other FMT-IBS studies have defined a 75 reduction in IBS-SSS as the definition of response, making it difficult to compare the results of interventions between studies.^18,45^ We urge future studies to report full IBS-SSS in addition to clearly defined definitions of clinical response.

### Perspectives

Based on the knowledge that FMT capsules induce long-term changes in the recipients’ microbiomes, future trials should utilize third-generation sequencing technologies to assess the functional relevance of changes to recipients’ microbiomes and compare these to changes with placebo patients. This could also be used to investigate whether parts of the microbiota other than bacteria and archaea, such as fungi, viruses, parasites and bacteriophages drive potential beneficial effects, which have been proposed as the driving mechanism in the treatment of other diseases through FMT.^47^

Since the importance of the microbiome in the etiology of IBS is not fully understood and patients are heterogeneous, future IBS-trials should possibly try to address IBS patients who would most possibly benefit from FMT. A recently published RCT by Holvoet et al.^42^ only including IBS patients with predominantly bloating reported beneficial change in symptoms in the group receiving FMT. Furthermore, patients with post-infectious IBS, where a known pathogen and/or the treatment thereof (e.g. with antibiotics) could disturb the microbiome, could possibly be a group that could benefit the most from FMT treatment as indicated by a sub-group analysis in the study by Aroniadis et al.^19^

## Conclusion

Twelve days of treatment with FMT capsules induces significant short- and long-term changes in the recipients’ microbiomes for up to 6 months. The microbiomes of FMT recipients both receive bacteria, including strictly anaerobic oxygen-sensitive bacteria from the donors and also lose bacteria. The changes in the gut microbiome of the FMT recipients were not correlated with clinical improvements. The role of the gut microbiome in IBS still remains unsolved and future studies are needed to access whether changing the microbiome can be correlated with beneficial effects in patients with IBS.

## Materials and methods

### Patient selection and treatment groups

Fifty-two adult patients with IBS were included into a double-blinded placebo-controlled clinical trial. Patients were randomly assigned to the FMT group or the placebo group. All patients underwent an oral bowel cleansing (Moviprep®) before starting a twelve-day course of 25 FMT or placebo capsules. The FMT capsules contained a fecal preparation mixed from four different donors where one dose (25 capsules) contained approximately 12 g of fecal preparation derived from 50 g fresh feces. Patients provided stool samples at a baseline clinical visit and at follow-up visits 2 weeks, 1 month, 3 months and 6 months after inclusion. During these clinical visits, IBS severity was assessed using the IBS-SSS.^23^ Full details regarding patient selection, donor selection, the clinical intervention, FMT capsule preparation and an ethics statement are available in the report focusing on the clinical aspects of this trial.^21^

Of the 52 IBS patients, 22 patients in the FMT group and 24 patients in the placebo group were evaluated at the primary endpoint after 3 months. Eight and 19 patients in the FMT and placebo groups, respectively, exhibited a clinical response, defined a priori as a decrease of 50 points in the IBS-SSS compared to baseline.^21^

### Sequencing data

The data in this study were produced in a related study primarily documenting the clinical results^21^ using Illumina’s MiSeq platform to sequence V3-V4 16S rRNA amplicons derived from fecal samples. The sequencing data were screened for human reads using bowtie2^48^ with the switches `-k 1` and `–very-sensitive-local` and reads with matches were removed from the analysis. All remaining reads were uploaded to the SRA under BioProject number PRJNA679690.

### Primary sequence analysis

Sequencing data were processed to produce a feature table of ASVs, ASV sequences and a phylogenetic tree of ASV sequences as previously described elsewhere^21^ except that the clustering step was performed with unoise3^49^ instead of uparse.^50^

### Microbiota analysis

The feature table was converted into biom format using biom.^51^ The feature table, ASV sequences and ASV tree were used as input to the qiime v1.9.1 core diversity analysis pipeline^52^ to derive measures of alpha diversity (Chao1) and beta-diversity (weighted and unweighted unifrac distances).^53^ Similarly, the Rhea pipeline^54^ was used to derive further estimators of alpha-diversity (Shannon, Effective Shannon, Simpson and Effective Simpson) and beta-diversity (generalized unifrac). Ordination was performed using the stats.ordination.pcoa function of python’s skbio library v0.5.6. Student’s t-test, the Mann–Whitney U-test and Spearman’s rank correlation were performed using the versions in python’s scipy library (v1.3.3). The Benjamini–Hochberg method was used for false-positive rate control as implemented in (https://github.com/padbr/asat/blob/master/benjamini_hochberg_p_adjust.py). Student’s t-test was used to compare sample means for alpha-diversity, while the non-parametric Mann-Whitney U-test was used to compare distributions of beta-diversity distances and ASV relative abundances. Potential correlations between IBS scores and microbiome data were assessed using Spearman’s rank correlation.

### Grouping by initial diversities

Where patients were split into groups based on the initial diversities (Chao1) of their fecal microbiota, normal initial diversity was defined as greater than or equal to the mean minus one standard deviation of the donors’ diversities. Low initial diversity was defined as less than the mean minus three standard deviations of the donors’ diversities. These definitions of normal and low initial diversity intentionally leave a “grey” zone in between to avoid the chance of classifying patients with highly similar initial diversities in opposite groups.

### Calculating gain and loss scores

For each ASV in each patient’s time-course, it was independently assessed if that ASV was gained or lost following treatment. For clarity, here, the gain of an ASV means that it is absent before treatment and consistently present following treatment, and vice versa for the loss of an ASV. In doing so, it is necessary to consider whether an apparent gain or loss could be explained by sampling disparities (e.g. to rule out that the absence of an ASV is not due to low sample coverage). It is also necessary to consider if putatively gained or lost ASVs are consistently present or absent in the follow-up time-points in order to rule out cases where certain ASVs are present transiently and not gained or lost due to the treatment.

In the cases where an ASV’s abundance at the initial time-point (T_0_) is zero, then a gain score is assessed (and the corresponding loss score is set to 0). An expected proportion is calculated by multiplying the sample coverage at T_0_ by the average proportion of the ASV seen in the follow-up time points. The survival function of python’s scipy.stats.poisson library is called on the expected frequency, giving a score that is used as a probability that the absence is not explicable by low sample coverage. Then the ASV’s abundances were assessed at the follow-up time-points (2 weeks, 1 month, 3 months and 6 months) in order to determine if the ASV is consistently present in sufficiently high proportions to indicate that the ASV was present in all post-treatment time-points. To do this, each pairwise set of two chronologically adjacent time-points were assessed in series. If the ASV is present in both time-points, a score of 1 is returned. If it is absent at both times, a score of 0 is returned. If the ASV is present in 1 and absent in the other, then an expected observation count is calculated for the absent sample by multiplying the sample coverage by the proportion in the other sample of the pair and again using the survival function to calculate the probability that the absence can be explained by low sample coverage. It is the probability that is returned as the score. All adjacent scores in the series and multiplied pairwise, and an average of the resulting products is multiplied by the probability that the initial absence was not due to low coverage.

In the cases where an ASVs abundance at T_0_ in a patient is greater than zero, then a loss score is derived (and the gain score is effectively set to 0). The following time-points are examined in chronological order. If the ASV is observed in the sample for a given time-point it was assigned a loss score of 0. If it was not observed in a sample, an expected observation count was calculated by multiplying the sample’s coverage by the ASV’s relative abundance at T_0_. Using the expected observation count, the survival function is used to return a probability that the ASV was not seen due to low sample coverage, which is then returned as a loss score. All pairwise adjacent loss scores were multiplied by each other and it is the average of these products that is returned as the ASV’s loss score for that patient. A cutoff of 0.85 was applied to the gain and loss scores. The gain and loss scores were calculated as described using a python script available at https://github.com/padbr/asat/blob/master/expected_intransience.poisson.py. A patient by ASV feature-table was made, where lost ASVs were represented with 0, unchanged ASVs were represented as 1 and gained ASVs were represented as 2. Based on this, a Euclidean distance matrix was calculated and used for principle coordinates analysis using python’s skbio library.

### Assessing engraftment using sourcetracking software

Sourcetracker2^27^ uses a Bayesian algorithm to estimate the proportions of a sink-microbiome that can be attributed to originating from various source-microbiomes. It was run with mapfiles specifying each individual donor as a separate source and with the ‘–per_sink_feature_assignments’ flag to also get a feature table detailing how much each source contributed to each ASV in the sink. These values (how much each sink contributed for each ASV) were used to calculate proportions for which each ASV was attributed to coming from the donors or to coming from the patients’ pre-treatment microbiota. For calculating such proportions for taxonomic groupings, the average of all of the proportions for each ASV assigned to that taxonomic classification was used. Histograms of the proportions of ASVs assigned to particular lineages inferred to have originated from the donors’ microbiota indicate that there is some noise in the data (Supplementary file, figure 6), with lineage-dependent variations in this noise. For this reason, when assessing what proportion of each taxonomy term is likely to have originated from the donors’ microbiota, the placebo groups’ proportions estimated to have come from the donors were used as a measure of background. If more of an ASV or taxonomic grouping was classed as coming from the donors in the placebo group, the estimated proportion coming from the donor in the FMT groups was set to zero. Otherwise, a new proportion was calculated by (P_FMT_ – P_Placebo_)/(1 – P_Placebo_). For plotting on a tree, the rescaled proportions were colored using python’s matplotlib.colormap module and drawn using biopython’s Phylo.tree.draw function.

## Supplementary Material

Supplemental Material

## Data Availability

All non-human sequence data were uploaded to the Sequence Read Archive (SRA) under BioProject number PRJNA679690.

## Clinical trial identifier

The study was registered in ClinicalTrials.gov (NCT02788071)
