# Supplementary material for: Engraftment of strictly anaerobic oxygen-sensitive bacteria in irritable bowel syndrome patients following fecal microbiota transplantation does not improve symptoms
Source: Gut Microbes. 2021 Jun 1;13(1):1927635. doi: 10.1080/19490976.2021.1927635 (PMC8183560; doi:10.1080/19490976.2021.1927635)
Supplement: Supplemental Material [file KGMI_A_1927635_SM8184.zip › Supplementary information/Supplementary_file_1.docx]

Supplementary file 1. Engraftment of strictly anaerobic oxygen-sensitive bacteria in irritable bowel syndrome patients following fecal microbiota transplantation does not improve symptoms

Table 1a: Mean fecal α-diversity (CHAO1-richness) in the treatment groups

|  | FMT (n=22) | Placebo  (n=24) | Difference in change from baseline* |
| --- | --- | --- | --- |
| Baseline | 550.6 | 587.1 |  |
| 15 days | 680.6  p=0.0002 | 581.1  p=0.9492 | 129.6  p=0.0017 |
| 1 month | 665.0  p=0.0022 | 607.1  p=0.1853 | 94.8  p=0.0246 |
| 3 months | 732.1  p<0.0001 | 617.9  p=0.1481 | 151.0  p<0.0001 |
| 6 months | 675.5  p=0.0002 | 614.1  p=0.4572 | 103.3  p=0.0125 |

Paired t-tests (p≤0.05) were used to compare α-diversity levels at each time-point after baseline with the levels at baseline. n=number of patients. * The difference in change from baseline column was calculated as the average difference of the FMT patients from their respective baseline α-diversities, minus that of the placebo group in order to estimate the effect of the microbiological content of the FMT capsules on α-diversity.

**Table 1b.** **Comparisons of diversity with baseline**

| Label | Time | N | Chao1 | | | ShannonE | | | SimpsonE | | |
| --- | --- | --- | --- | --- | --- | --- | --- | --- | --- | --- | --- |
|  |  |  | T0 | TT | Pval | T0 | TT | Pval | T0 | TT | Pval |
| FMT Patients | 0.5 | 16 | 549.35 | 680.59 | 0.0002 | 94.36 | 111.98 | 0.0937 | 30.61 | 36.6 | 0.3177 |
| FMT Patients | 1 | 19 | 539.49 | 664.97 | 0.0022 | 89.7 | 112.61 | 0.0988 | 29.3 | 36.74 | 0.1915 |
| FMT Patients | 3 | 21 | 550.64 | 732.07 | 0 | 93.37 | 152.05 | 0 | 30.37 | 53.45 | 0.0003 |
| FMT Patients | 6 | 21 | 550.64 | 675.46 | 0.0002 | 93.37 | 132.33 | 0.0014 | 30.37 | 50.23 | 0.0008 |
| Placebo Patients | 0.5 | 22 | 579.49 | 581.14 | 0.9492 | 105.38 | 92.35 | 0.3192 | 37.91 | 31.41 | 0.307 |
| Placebo Patients | 1 | 22 | 576.41 | 607.09 | 0.1853 | 103.01 | 110.58 | 0.5792 | 36.6 | 39.38 | 0.6754 |
| Placebo Patients | 3 | 23 | 587.43 | 617.89 | 0.1481 | 106.27 | 112.78 | 0.518 | 38.03 | 41.04 | 0.5649 |
| Placebo Patients | 6 | 23 | 592.53 | 614.07 | 0.4572 | 109.55 | 116.85 | 0.5608 | 39.08 | 41.71 | 0.6569 |
| FMT Responders | 0.5 | 5 | 596.52 | 677.81 | 0.2668 | 111.86 | 96.23 | 0.3064 | 39.26 | 30.18 | 0.2221 |
| FMT Responders | 1 | 8 | 585.21 | 637.31 | 0.3981 | 103.77 | 103.76 | 0.9996 | 35.94 | 36.2 | 0.9825 |
| FMT Responders | 3 | 8 | 585.21 | 725.16 | 0.0051 | 103.77 | 149.31 | 0.0174 | 35.94 | 52.49 | 0.0468 |
| FMT Responders | 6 | 8 | 585.21 | 691.62 | 0.0068 | 103.77 | 130.29 | 0.1836 | 35.94 | 48.97 | 0.2279 |
| FMT Non-responders | 0.5 | 11 | 527.91 | 681.86 | 0.0002 | 86.41 | 119.13 | 0.0098 | 26.68 | 39.51 | 0.1024 |
| FMT Non-responders | 1 | 11 | 506.23 | 685.09 | 0.0008 | 79.47 | 119.05 | 0.0048 | 24.48 | 37.13 | 0.0129 |
| FMT Non-responders | 3 | 13 | 529.37 | 736.32 | 0 | 86.98 | 153.74 | 0.0011 | 26.94 | 54.04 | 0.0036 |
| FMT Non-responders | 6 | 13 | 529.37 | 665.52 | 0.0061 | 86.98 | 133.58 | 0.0037 | 26.94 | 51 | 0.0008 |
| Placebo Responders | 0.5 | 17 | 585.5 | 565.18 | 0.3867 | 105.71 | 89.6 | 0.1405 | 36.71 | 30.25 | 0.2349 |
| Placebo Responders | 1 | 17 | 581.51 | 605.89 | 0.2168 | 102.64 | 112.54 | 0.2918 | 35.01 | 40.48 | 0.2401 |
| Placebo Responders | 3 | 18 | 595.31 | 622.1 | 0.1212 | 106.82 | 119.08 | 0.1717 | 36.94 | 44.16 | 0.1552 |
| Placebo Responders | 6 | 18 | 601.82 | 605.75 | 0.8917 | 111.01 | 118.26 | 0.5245 | 38.27 | 41.96 | 0.4969 |
| Placebo Non-responders | 0.5 | 5 | 559.06 | 635.42 | 0.391 | 104.27 | 101.71 | 0.9596 | 41.99 | 35.37 | 0.7852 |
| Placebo Non-responders | 1 | 5 | 559.06 | 611.14 | 0.5551 | 104.27 | 103.9 | 0.995 | 41.99 | 35.64 | 0.8208 |
| Placebo Non-responders | 3 | 5 | 559.06 | 602.75 | 0.6104 | 104.27 | 90.12 | 0.7064 | 41.99 | 29.83 | 0.4725 |
| Placebo Non-responders | 6 | 5 | 559.06 | 644.04 | 0.3631 | 104.27 | 111.8 | 0.8735 | 41.99 | 40.78 | 0.9561 |

Groups of samples are compared at each post-treatment time-point (TT) with their baseline diversities (T0). P-values (Pval) shown are the result of 2-tailed paired T-tests. ShannonE and SimpsonE represent the number of species at equal abundance required to have the same Shannon diversity or Simpson diversity, respectively, as the samples. Due to missing samples and uneven coverage there can be disparities between the number of paired samples at each time-point within each group (label).

**Table 2.** **Generalized unifrac distances of the patients to the donors (Beta diversity) in the treatment groups at each time-point**

|  | 0.5 | 1 | 3 | 6 |
| --- | --- | --- | --- | --- |
| FMT Patients | N=16  BL: 0.619 (0.104)  TP: 0.595 (0.088)  P=0.0894 | N=19  BL: 0.632 (0.102)  TP: 0.593 (0.122)  P=0.0008 | N=21  BL: 0.626 (0.101)  TP: 0.560 (0.085)  P=0.0000 | N=21  BL: 0.626 (0.101)  TP: 0.587 (0.102)  P=0.0014 |
| Placebo Patients | N=22  BL: 0.598 (0.104)  TP: 0.607 (0.081)  P=0.4100 | N=22  BL: 0.601 (0.102)  TP: 0.600 (0.077)  P=0.3755 | N=23  BL: 0.602 (0.100)  TP: 0.627 (0.084)  P=0.0007 | N=23  BL: 0.597 (0.104)  TP: 0.596 (0.082)  P=0.4361 |
| FMT Responders | N=5  BL: 0.579 (0.102)  TP: 0.585 (0.077)  P=0.8228 | N=8  BL: 0.610 (0.095)  TP: 0.609 (0.139)  P=0.7506 | N=8  BL: 0.610 (0.095)  TP: 0.552 (0.081)  P=0.0028 | N=8  BL: 0.610 (0.095)  TP: 0.564 (0.095)  P=0.0151 |
| FMT Non-responders | N=11  BL: 0.637 (0.100)  TP: 0.599 (0.092)  P=0.0222 | N=11  BL: 0.647 (0.103)  TP: 0.581 (0.106)  P=0.0000 | N=13  BL: 0.635 (0.103)  TP: 0.564 (0.087)  P=0.0000 | N=13  BL: 0.635 (0.103)  TP: 0.601 (0.104)  P=0.0251 |
| Placebo Responders | N=17  BL: 0.595 (0.104)  TP: 0.619 (0.078)  P=0.0659 | N=17  BL: 0.599 (0.101)  TP: 0.604 (0.080)  P=0.0607 | N=18  BL: 0.600 (0.099)  TP: 0.630 (0.083)  P=0.0006 | N=18  BL: 0.594 (0.103)  TP: 0.601 (0.082)  P=0.2863 |
| Placebo Non-responders | N=5  BL: 0.608 (0.106)  TP: 0.567 (0.078)  P=0.1169 | N=5  BL: 0.608 (0.106)  TP: 0.586 (0.064)  P=0.4553 | N=5  BL: 0.608 (0.106)  TP: 0.617 (0.084)  P=0.3317 | N=5  BL: 0.608 (0.106)  TP: 0.578 (0.078)  P=0.9108 |

Average generalized unifrac distances of all patients in each group to all donors are shown with standard deviations in parentheses. Post treatment time-points are indicated in the top row and indicate the number of months since inclusion. N: indicates the numbers of patients for whom distance data at baseline and at the indicated time-point was available, BL: the average distances of the patients to the donors at baseline; TP: the average distances of the patients to the donors at the time-point indicated in the first row; P: indicates the probability that the baseline distances and the time-point distances come from the same distribution.

**Table 3. Change in abundance of certain ASVs in treatment groups at different time-points**

| **Group1** | **Group2** | **Baseline** | **2 weeks** | **1 month** | **3 months** | **6 months** |
| --- | --- | --- | --- | --- | --- | --- |
| Responders | Non-responders | 0 / 0 | 0 / 0 | 0 / 0 | 0 / 0 | 0 / 0 |
| FMT | Placebo | 0 / 0 | 64 / 2 | 63 / 10 | 99 /7 | 59 / 12 |
| FMT responders | FMT non-responders | 0 / 0 | 0 / 0 | 0 / 0 | 0 / 0 | 0 / 0 |
| FMT responders | Placebo responders | 0 / 0 | 74 / 0 | 60 / 5 | 58 / 2 | 66 / 5 |
| Placebo responders | Placebo non-responders | 0 / 0 | 0 / 0 | 0 / 0 | 0 / 0 | 0 / 0 |
| FMT non-responders | Placebo non-responders | 0 / 0 | 0 / 0 | 0 / 0 | 0 / 0 | 0 / 0 |

False discovery rate (FDR) controlled differentially abundant amplicon sequence variants (ASVs) between Group1 and Group2. The numbers indicate (number more abundant in Group1) / (number more abundant in Group2). FMT, Fecal Microbiota Transplantation

**Figure 1a. Correlation between diversity (CHAO-1 richness) and symptoms (IBS-SSS)**

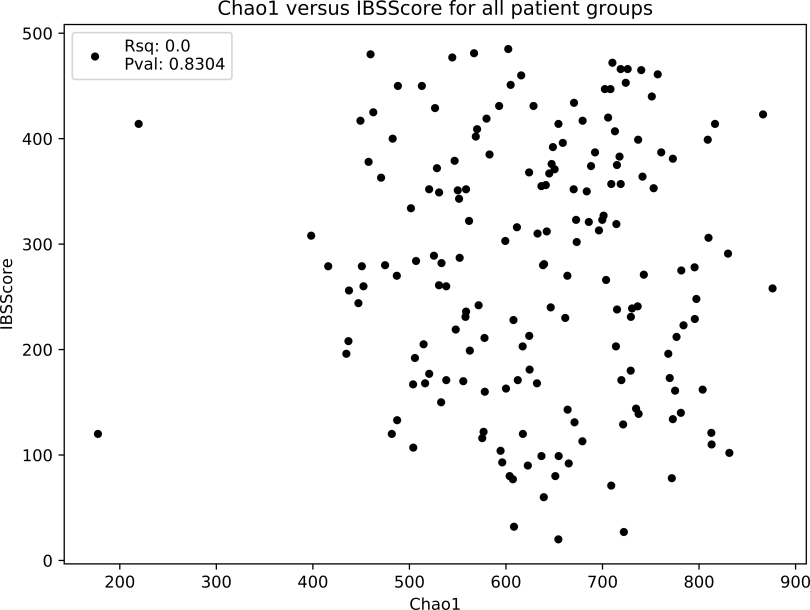

Diversity (Chao1) and IBS-SSS are plotted for all patients. Rsq values are squared Pearson correlation coefficients and P-values indicate the probability that there is no significant correlation between diversity (Chao1) and IBS-SSS.

**Figure 1b. Correlation between change in diversity (CHAO-1 richness) and symptoms (IBS-SSS) in the treatment groups**


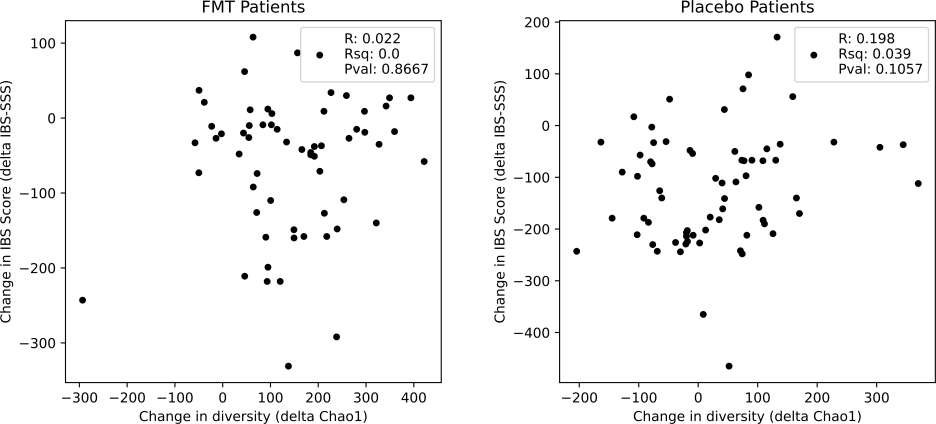


Change in Chao1 from baseline to all post-baseline samples versus change in IBS-SSS from baseline to all post-baseline samples. Rsq values are squared Pearson correlation coefficients and P-values indicate the probability that there is no significant correlation between diversity (Chao1) and IBS-SSS

**Figure 2. ASVs with altered abundance at different time-points in treatment groups**

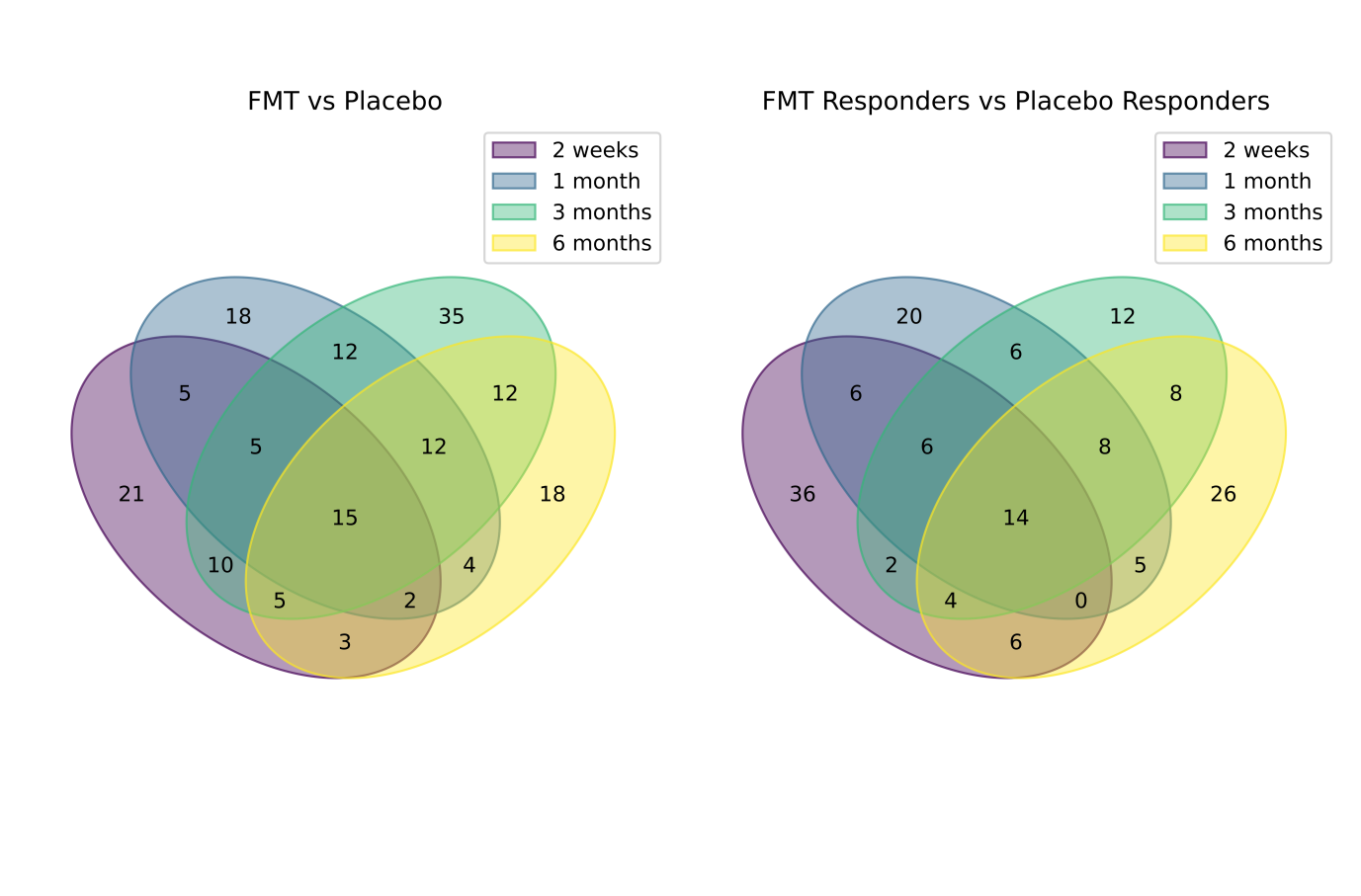


Venn diagrams showing the overlap in numbers of ASVs significantly altered in abundances at each time-point between treatment groups and between FMT and Placebo responders. 14 and 15 ASVs were altered at all time-points for the two respective comparisons, FMT vs Placebo and FMT responders vs Placebo responders.

**Figure 3.** **Heat map of sequence depth of all patients at each time-point.**

Heat map indicating the numbers of paired reads mapped to ASVs for donors and each patient at each time-point. Note that the time-points (y-axis) do not apply to the donors, whose fecal samples were sequenced once only, and the white square at the bottom of the donors’ column is because there were four donors (and five time-points)

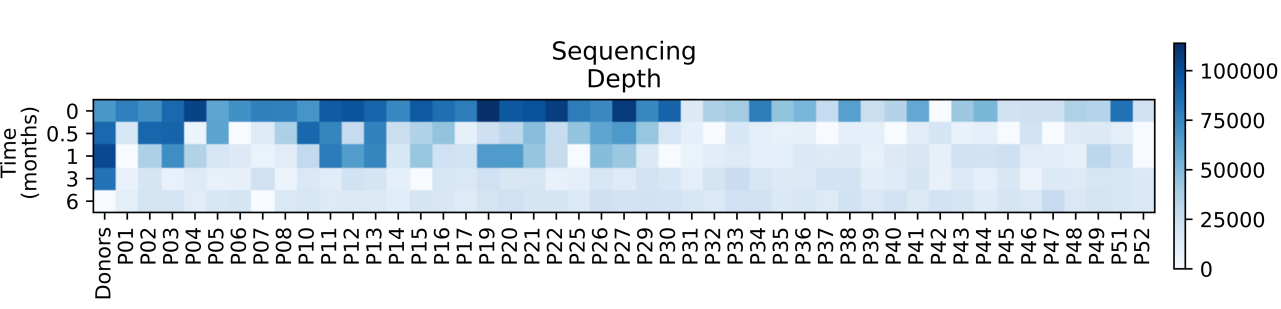


**Figure 4. Difference in gain and loss profile of treated patients.**

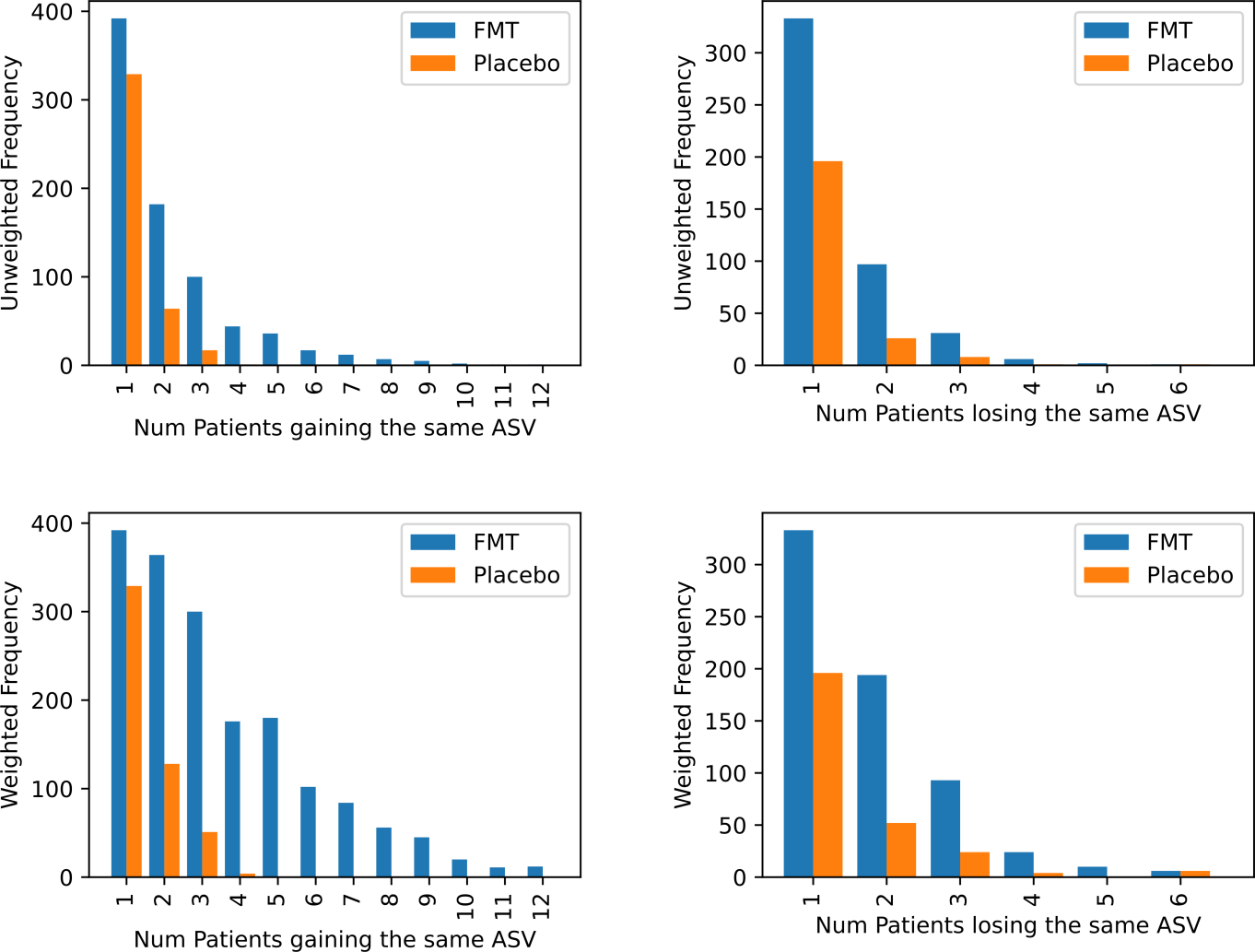


The numbers of gains and losses are presented in unweighted and weighted formats. Unweighted numbers are a simple count of ASVs that are either gained or lost, without regard for the number of patients in which the ASVs were gained or lost. The weighted numbers are the product of the number of ASVs gained or lost and the number of patients in which they were gained or lost.

**Figure 5. Gain and loss on an individual patient level**


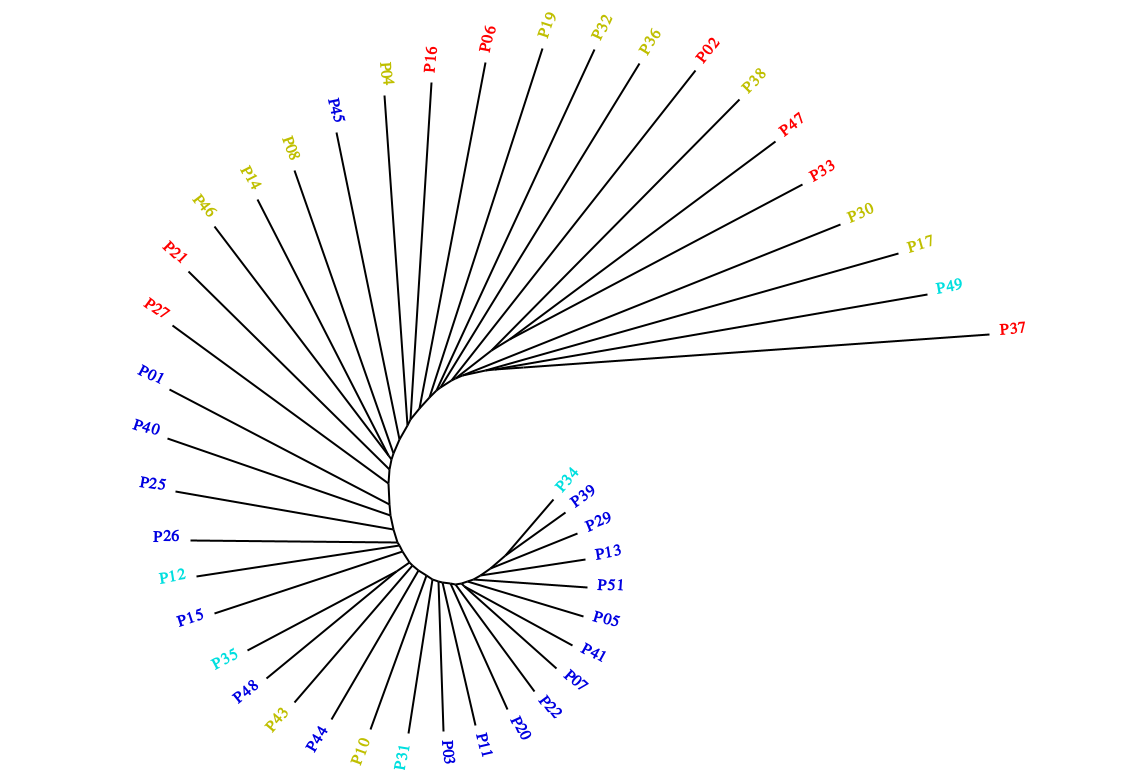
Gain and Loss WPGMA: Based on a Euclidean distance matrix, a tree was constructed based on the weighted pair group method with arithmetic mean. The labels of the terminal nodes represent patients where the coloring indicates treatment and response: red – FMT responders, mustard yellow – FMT non-responders, blue – Placebo responders, cyan – Placebo non-responders.

**
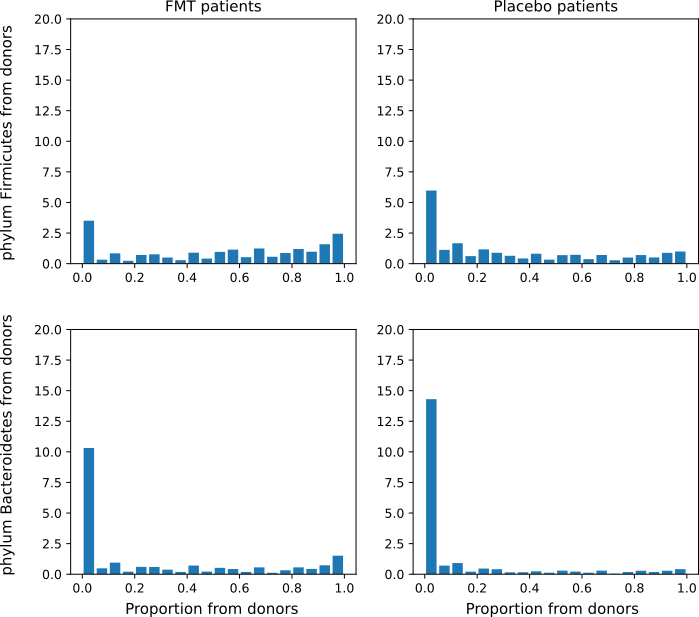
Figure 6**. **Particular lineages to have originated from the donors**

Histogram of particular lineages to have originated from the donors. The proportions (on a scale of 0 to 20), assessed to have come from the donors’ microbiotas, of each ASV assigned to either the Firmicutes or Bacteroidetes in each patient were plotted in a histogram. The relatively smaller heights of the bars on the left and the relative larger heights on the right for the FMT patients indicate that engraftment from the donors is occurring above background levels. It cannot be concluded that Firmicutes engraft more than Bacteroidetes in the FMT patients as it appears here, because the same relative trend is evident in the Placebo patients.
